# From traditional metabolic markers to ensemble learning: comparative application of machine learning models for predicting NAFLD risk in adolescents

**DOI:** 10.3389/fendo.2025.1681686

**Published:** 2025-10-29

**Authors:** Chenming Zhang, Bin Niu, Rong Wang, Liaoyun Zhang

**Affiliations:** 1 Academy of Medical Sciences, Shanxi Medical University, Taiyuan, China; 2 Department of Infectious Diseases, The First Hospital of Shanxi Medical University, Taiyuan, China; 3 Graduate School, Shanxi Medical University, Taiyuan, China

**Keywords:** machine learning, non-alcoholic fatty liver disease, adolescents, feature selection, public health

## Abstract

**Background:**

Non-alcoholic fatty liver disease (NAFLD) is increasingly prevalent among adolescents and poses a significant public health challenge. Due to limitations in imaging and invasive diagnostic methods such as liver biopsy, there is a pressing need for accurate, cost-effective, and non-invasive risk prediction tools. This study aims to develop and compare multiple machine learning (ML) models to predict NAFLD risk in adolescents using routine anthropometric and laboratory data from the National Health and Nutrition Examination Survey (NHANES) 2011–2020 dataset.

**Methods:**

Data from 2,132 U.S. adolescents (NHANES 2011–2020) were analyzed. Nine machine learning (ML) models were developed using features selected by Light Gradient Boosting Machine (LightGBM). Performance was assessed by AUC, accuracy, sensitivity, precision, F1-score, and calibration. The Extra Trees (ET) model was further compared with TyG-based logistic regression models. Model interpretability was evaluated using SHapley Additive exPlanations (SHAP), and an interactive online prediction tool was deployed.

**Results:**

NAFLD prevalence was 13.0%. The ET model achieved the best overall performance (AUC = 0.784, ACC = 0.773, Kappa = 0.320), outperforming other ML algorithms and TyG-based models, which showed higher sensitivity but poorer precision. SHAP analysis identified waist circumference, triglycerides, insulin, and HDL as key predictors, revealing nonlinear threshold effects. The online tool allows individualized risk estimation based on routine clinical variables.

**Conclusion:**

The ET-based ML model provides an accurate and interpretable approach for adolescent NAFLD risk prediction. By surpassing traditional metabolic indicators and offering an accessible web-based calculator, it supports scalable, cost-effective early screening and targeted prevention strategies.

## 1 Introduction

Non-alcoholic fatty liver disease (NAFLD) is characterized by excessive hepatic fat accumulation and is closely associated with insulin resistance, histologically defined as steatosis in more than 5% of hepatocytes (1). NAFLD has become a leading cause of chronic liver disease worldwide, with an estimated global prevalence of 32% (40% in males and 26% in females) (2). In 2024, the European Association for the Study of the Liver (EASL) recommended replacing the term NAFLD with metabolic dysfunction–associated steatotic liver disease (MASLD) (3). However, because the NHANES dataset and most prior epidemiological studies still adopt NAFLD, this terminology is retained in the present study. The prevalence of NAFLD has been reported to reach nearly 70% among overweight individuals (4), N and while disease progression is often slow, it can lead to fibrosis, cirrhosis, hepatocellular carcinoma, or end-stage liver disease in a subset of patients (5). I In recent years, pediatric NAFLD has risen in parallel with the global obesity epidemic, highlighting the urgent need for early detection and prevention strategies (6).

Despite its growing incidence, there is no consensus on standardized diagnostic criteria for NAFLD in adolescents. Liver biopsy remains the diagnostic gold standard but is invasive and unsuitable for large-scale use (7). Non-invasive imaging techniques, such as vibration-controlled transient elastography (VCTE), point shear wave elastography (pSWE), two-dimensional shear wave elastography (2D-SWE), and magnetic resonance elastography (MRE), as well as MRI-based methods (e.g., corrected T1 mapping, diffusion-weighted imaging), offer promising alternatives but face limitations in cost, availability, and pediatric accuracy (8).

Parallel to advances in imaging, artificial intelligence (AI) has gained prominence in healthcare, with machine learning (ML) enabling more objective risk prediction and individualized treatment strategies (9, 10). Traditional NAFLD risk assessment often relies on clinician judgment or simple indices, which may lack precision. In this context, the triglyceride–glucose (TyG) index and its derivatives have been proposed as surrogate markers of insulin resistance, showing predictive value in NAFLD and metabolic disorders (11, 12). However, these linear constructs are based on limited variables and may not capture the multifactorial, nonlinear nature of NAFLD. This gap highlights the promise of ML methods in refining risk prediction, particularly in youth populations.

Recent ML studies have developed predictive models for progression from NAFLD to more severe outcomes such as NASH, fibrosis, and hepatocellular carcinoma in adults (13, 14). Although some research has applied ML to adolescents, limitations remain, including reliance on complex predictors and lack of validation in real-world settings (15–17). Therefore, the present study aimed to identify key predictors using robust feature selection strategies and to develop an interpretable ML-based system for predicting adolescent NAFLD. By leveraging readily obtainable clinical and laboratory indicators, this approach provides a cost-effective and scalable tool to support early screening and intervention. Materials and methods.

## 2 Materials and methods

### 2.1 Data sources and study population

The National Health and Nutrition Examination Survey (NHANES) is a population-based cross-sectional survey of U.S. adults and children, publicly available for epidemiological and clinical research. Data from the 2011–2020 cycles were used, and individuals aged 11–20 years were included. Further details are available on the NHANES website (https://www.cdc.gov/nchs/nhanes/index.html).

Sociodemographic variables included age, sex, and race/ethnicity (Mexican American, Other Hispanic, Non-Hispanic White, Non-Hispanic Black, and other races). Anthropometric measures were height, weight, waist circumference (WC), and body mass index (BMI). Laboratory parameters included white blood cell count (WBC), red blood cell count (RBC), platelet count (PLT), hemoglobin (HB), glycated hemoglobin (HbA1c), total cholesterol (TC), triglycerides (TG), high-density lipoprotein (HDL), low-density lipoprotein (LDL), fasting glucose (GLU), and fasting insulin. NAFLD was defined as alanine aminotransferase (ALT) >26 IU/L in males and >22 IU/L in females, without viral hepatitis, consistent with prior NHANES-based studies (18, 19).

The NHANES protocol was approved by the National Center for Health Statistics (NCHS) Research Ethics Review Board. Written informed consent was obtained at the time of data collection. As only de-identified, publicly available data were used, no additional institutional approval was required.

### 2.2 Feature selection

The Light Gradient Boosting Machine (LightGBM) package in Python was first used to rank variables by importance, and the top 10 predictors were retained based on AUC contribution. Because LightGBM also served as a benchmark model, feature stability was confirmed with a consensus strategy combining L1-penalized logistic regression, Boruta, and permutation importance. Six predictors (WC, TG, insulin, GLU, weight, BMI) were consistently identified, reducing bias toward tree-based methods (**Supplementary Figures S1**, **S2**).

### 2.3 Model construction, evaluation and validation

Using Python libraries including scikit-learn, XGBoost, and LightGBM, we constructed and evaluated nine supervised algorithms: artificial neural network (ANN), decision tree (DT), Extra Trees (ET), gradient boosting (GB), k-nearest neighbors (KNN), LightGBM, random forest (RF), support vector machine (SVM), and XGBoost. To address class imbalance (13% NAFLD vs. 87% non-NAFLD), the Synthetic Minority Oversampling Technique (SMOTE) was applied to the training set within each fold. Hyperparameters were optimized via grid search with five-fold stratified cross-validation (**Supplementary Table S1**), and final models were retrained on the full training set. Model performance was assessed by discrimination, calibration, and clinical utility.

To compare with traditional indicators, three metabolic indices were included: the triglyceride-glucose index (TyG), TyG-BMI, and TyG-waist circumference (TyG-WC). The TyG index was calculated as:


$$TyG=ln(\frac{TG(mg/\text{dL})\times FPG(mg/\text{dL})}{2})$$

where TG is triglycerides and FPG is fasting plasma glucose. Each index was first used in logistic regression models, followed by multivariate models combining TyG and its derivatives. These were systematically compared with ET models trained on the same dataset.

For interpretability, SHapley Additive exPlanations (SHAP) values were used to quantify each feature’s contribution. SHAP identified the most influential predictors, revealed nonlinear effects, and enabled individualized risk profiling. To enhance accessibility, we developed a user-friendly online prediction tool using Streamlit (https://jd82bumajen97hthfgjsmr.streamlit.app/; source code available on GitHub: https://github.com/moresaying98/NAFLD-adolescence). The interface follows the logic of SHAP force plots: after inputting individual anthropometric and laboratory values, it outputs a personalized NAFLD risk probability along with feature-specific contributions for intuitive interpretation.

### 2.4 Statistical analysis

Statistical analyses were performed using R (version 4.3.0) and Python (version 3.10.6). Normally distributed variables were expressed as mean ± standard deviation (SD) and compared using independent t-tests. Non-normally distributed variables were expressed as median (Q1, Q3) and compared with Mann–Whitney U tests. Categorical variables were presented as n (%) and compared using chi-square or Fisher’s exact tests. A two-sided p < 0.05 was considered statistically significant.

## 3 Results

**Figure 1:**
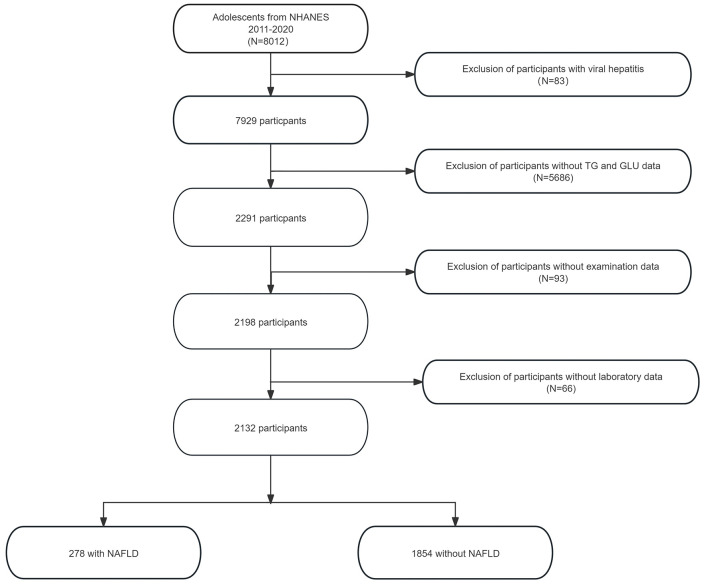
Flowchart of participant selection, showing exclusion criteria and the final sample of 2,132 adolescents used for model development.

After initial screening, 83 participants with viral hepatitis were excluded, leaving a preliminary sample of 7,929 individuals. Due to missing laboratory data, 5,638 participants were further excluded, reducing the sample size to 2,291. An additional 93 individuals were excluded due to incomplete physical examination data, resulting in 2,198 eligible participants. Finally, 66 more participants were excluded because of missing laboratory parameters, yielding a final analytic sample of 2,132 adolescents. Among them, 1,854 participants were identified as NAFLD-free, while 278 were diagnosed with NAFLD. A detailed flowchart of the participant selection process is presented in **Figure 1**.

A total of 2,132 participants were included in this study based on the inclusion criteria. Among them, 1,854 (86.96%) were classified as NAFLD-negative, while 278 (13.04%) were diagnosed with NAFLD. The baseline characteristics and group-wise comparisons are summarized in **Table 1**. Regarding anthropometric measures, participants in the NAFLD group had significantly higher height (167.69 ± 10.38 cm), weight [82.00 (65.10, 101.50) kg], BMI [28.50 (23.65, 34.68)], and waist circumference [94.10 (80.40, 110.45) cm] compared with those in the non-NAFLD group (all p < 0.001). In terms of hematological indicators, the NAFLD group showed significantly higher levels of white blood cell count [6.40 (5.30, 7.70)], red blood cell count [5.03 (4.72, 5.36)], hemoglobin [14.40 (13.40, 15.40)], and platelet count [257.50 (216.25, 293.00)] than the non-NAFLD group (all p < 0.05). In contrast, the HDL level was significantly lower in the NAFLD group compared to the non-NAFLD group [45.00 (39.00, 54.00) vs 52.00 (45.00, 61.00), p < 0.001]. Additionally, the NAFLD group exhibited significantly higher levels of TG, low-density LDL, TC, GLU, glycated HbA1c, and insulin than the non-NAFLD group (all p < 0.05). Among demographic variables, the proportion of males was significantly higher in the NAFLD group compared to the non-NAFLD group (63.67% vs. 49.24%, p < 0.001). Significant differences in racial/ethnic distribution were also observed between the two groups (p < 0.001).

**Table 1:** Baseline characteristics of participants stratified by NAFLD status.

Variables	Total (n = 2132)	0 (n = 1854)	1 (n = 278)	Statistic	*P*
Height cm, Mean ± SD	165.12 ± 9.93	164.73 ± 9.80	167.69 ± 10.38	t=-4.66	<.001
Age, M (Q_1_, Q_3_)	15.00 (13.00, 17.00)	15.00 (13.00, 17.00)	16.00 (14.00, 18.00)	Z=-4.74	<.001
Weight kg, M (Q_1_, Q_3_)	63.10 (52.80, 77.50)	61.55 (52.00, 73.88)	82.00 (65.10, 101.50)	Z=-12.53	<.001
BMI, M (Q_1_, Q_3_)	22.80 (19.90, 27.30)	22.20 (19.70, 26.20)	28.50 (23.65, 34.68)	Z=-12.52	<.001
WC, M (Q_1_, Q_3_)	78.50 (71.00, 90.20)	77.20 (70.40, 87.00)	94.10 (80.40, 110.45)	Z=-13.36	<.001
WBC, M (Q_1_, Q_3_)	6.10 (5.10, 7.30)	6.00 (5.00, 7.20)	6.40 (5.30, 7.70)	Z=-3.47	<.001
RBC, M (Q_1_, Q_3_)	4.83 (4.51, 5.15)	4.80 (4.48, 5.12)	5.03 (4.72, 5.36)	Z=-7.03	<.001
HGB, M (Q_1_, Q_3_)	13.90 (13.10, 14.90)	13.90 (13.00, 14.80)	14.40 (13.40, 15.40)	Z=-5.32	<.001
PLT, M (Q_1_, Q_3_)	247.00 (214.00, 286.00)	246.00 (214.00, 286.00)	257.50 (216.25, 293.00)	Z=-2.09	0.037
HDL, M (Q_1_, Q_3_)	51.00 (44.00, 60.00)	52.00 (45.00, 61.00)	45.00 (39.00, 54.00)	Z=-8.49	<.001
TG, M (Q_1_, Q_3_)	62.00 (44.00, 87.00)	60.00 (43.00, 83.75)	82.00 (55.25, 127.00)	Z=-9.15	<.001
LDL, M (Q_1_, Q_3_)	84.00 (69.00, 102.00)	83.00 (68.00, 100.00)	92.50 (72.00, 114.00)	Z=-4.93	<.001
TC, M (Q_1_, Q_3_)	152.00 (134.00, 172.00)	151.00 (133.25, 171.00)	160.00 (136.25, 183.00)	Z=-3.88	<.001
GLU, M (Q_1_, Q_3_)	95.00 (90.00, 100.00)	95.00 (90.00, 100.00)	97.00 (91.00, 102.00)	Z=-3.52	<.001
HbA1c, M (Q_1_, Q_3_)	5.30 (5.10, 5.50)	5.30 (5.10, 5.50)	5.30 (5.10, 5.50)	Z=-2.19	0.029
Insulin, M (Q_1_, Q_3_)	10.88 (7.28, 16.93)	10.29 (7.06, 15.48)	17.14 (10.28, 29.24)	Z=-10.32	<.001
Gender, n(%)				χ²=20.13	<.001
1	1090 (51.13)	913 (49.24)	177 (63.67)		
2	1042 (48.87)	941 (50.76)	101 (36.33)		
Race 4, n(%)				χ²=30.33	<.001
1	589 (27.63)	511 (27.56)	78 (28.06)		
2	566 (26.55)	516 (27.83)	50 (17.99)		
3	427 (20.03)	340 (18.34)	87 (31.29)		
4	550 (25.80)	487 (26.27)	63 (22.66)		

t, t-test; Z, Mann-Whitney test; χ², Chi-square test.

SD, standard deviation; M, Median; Q_1_, 1st Quartile; Q_3_, 3st Quartile.

Values are expressed as mean ± SD or median (Q_1_, Q_3_). Group comparisons were performed using t-test, Mann–Whitney U test, or Chi-square test. BMI, body mass index; WC, waist circumference; WBC, white blood cell count; RBC, red blood cell count; HGB, hemoglobin; PLT, platelet count; HDL, high-density lipoprotein; TG, triglyceride; LDL, low-density lipoprotein; TC, total cholesterol; GLU, fasting glucose; HbA1c, glycated hemoglobin.

**Figure 2:**
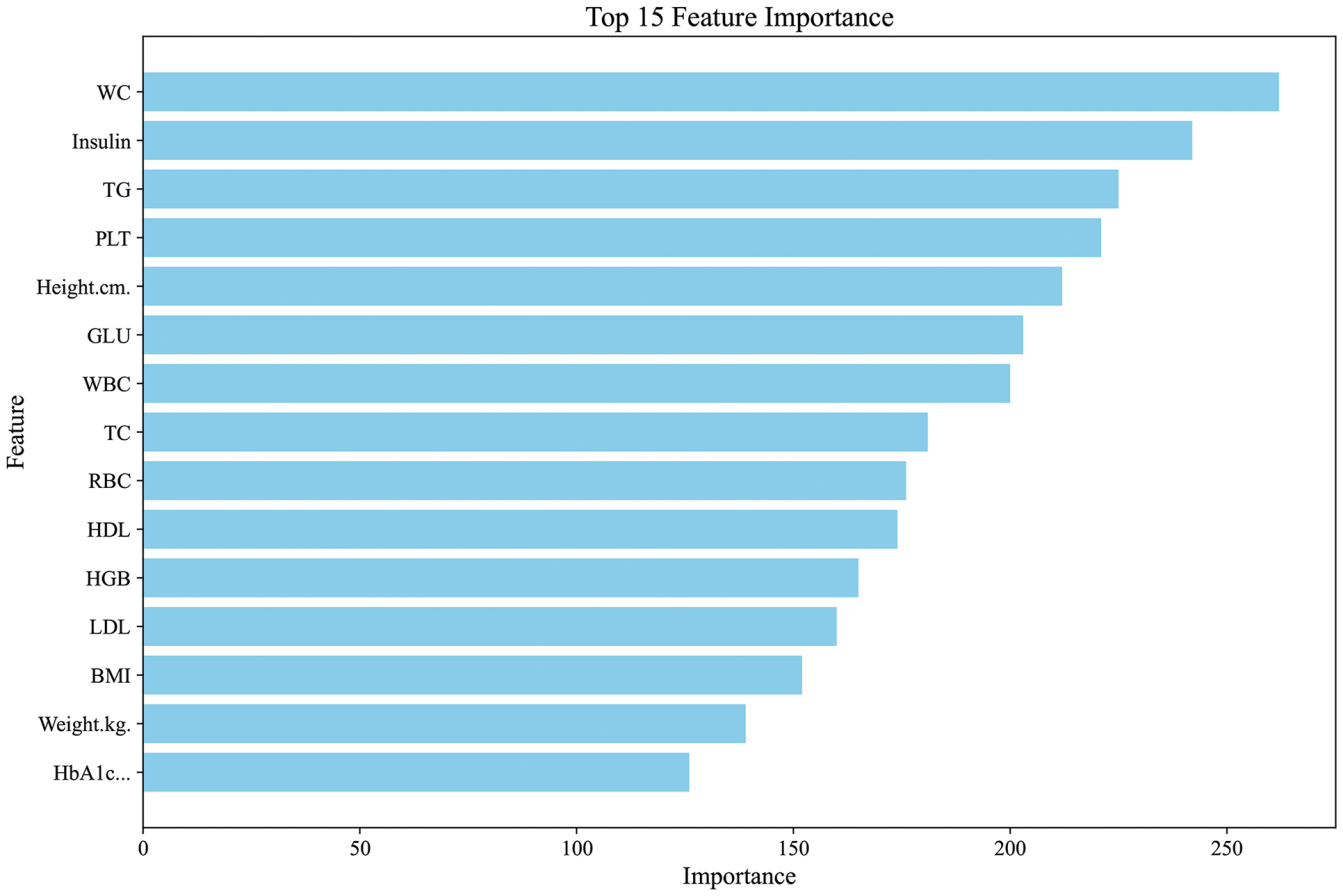
Variable importance ranking from the Light Gradient Boosting Machine (LGBM) model.

**Figure 3:**
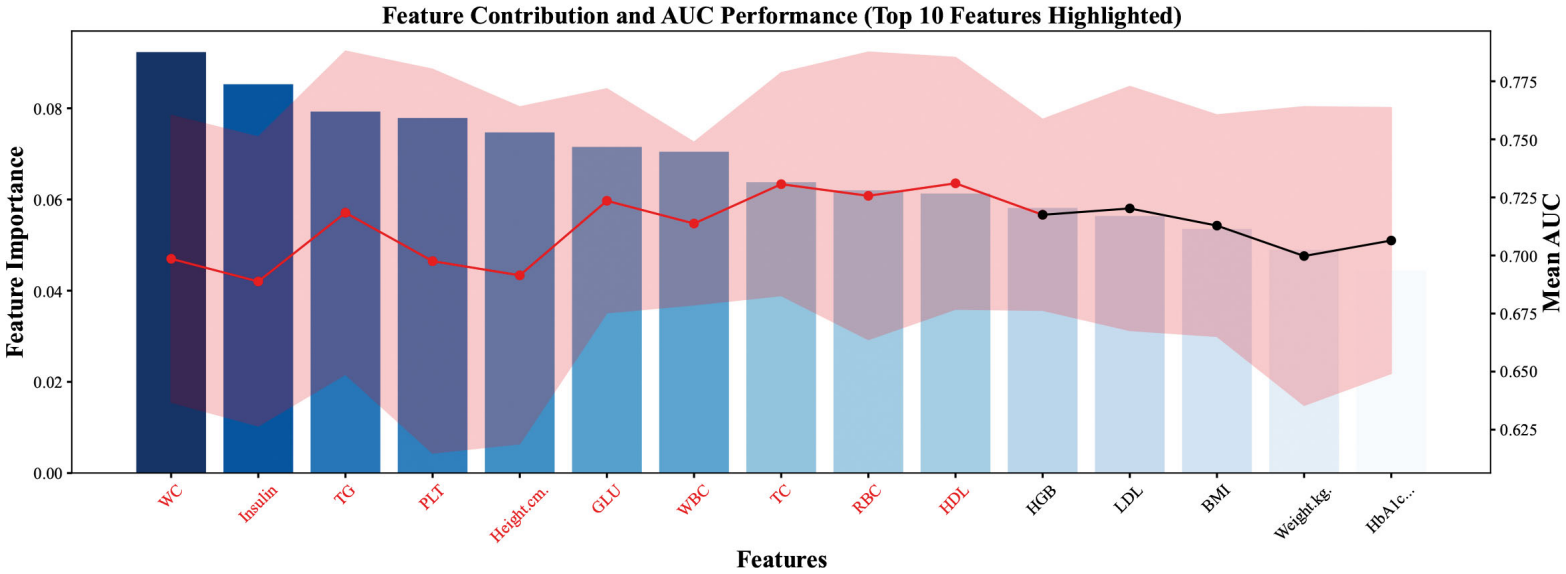
Feature selection using LGBM, with AUC plateauing after the 10th variable; top 10 predictors retained.

In this study, feature selection was performed using the LGBM algorithm implemented in Python. Initially, all variables were ranked based on feature importance scores derived from the initial LGBM classifier, and the top 15 variables were selected for further analysis (**Figure 2**). These variables were then sequentially added to the model in order of descending importance, and a series of LGBM classifiers were constructed to assess the incremental contribution of each variable to model performance. Model performance was evaluated using the (AUC). As shown in **Figure 3**, the AUC increased with the sequential addition of variables but plateaued after the inclusion of the 10th variable, indicating no substantial performance gain beyond that point. Therefore, the top 10 variables were selected for final model development. The final 10 key predictors included: WC, insulin, TG, PLT, Height, GLU, WBC, TC, RBC, and HDL.

**Figure 4:**
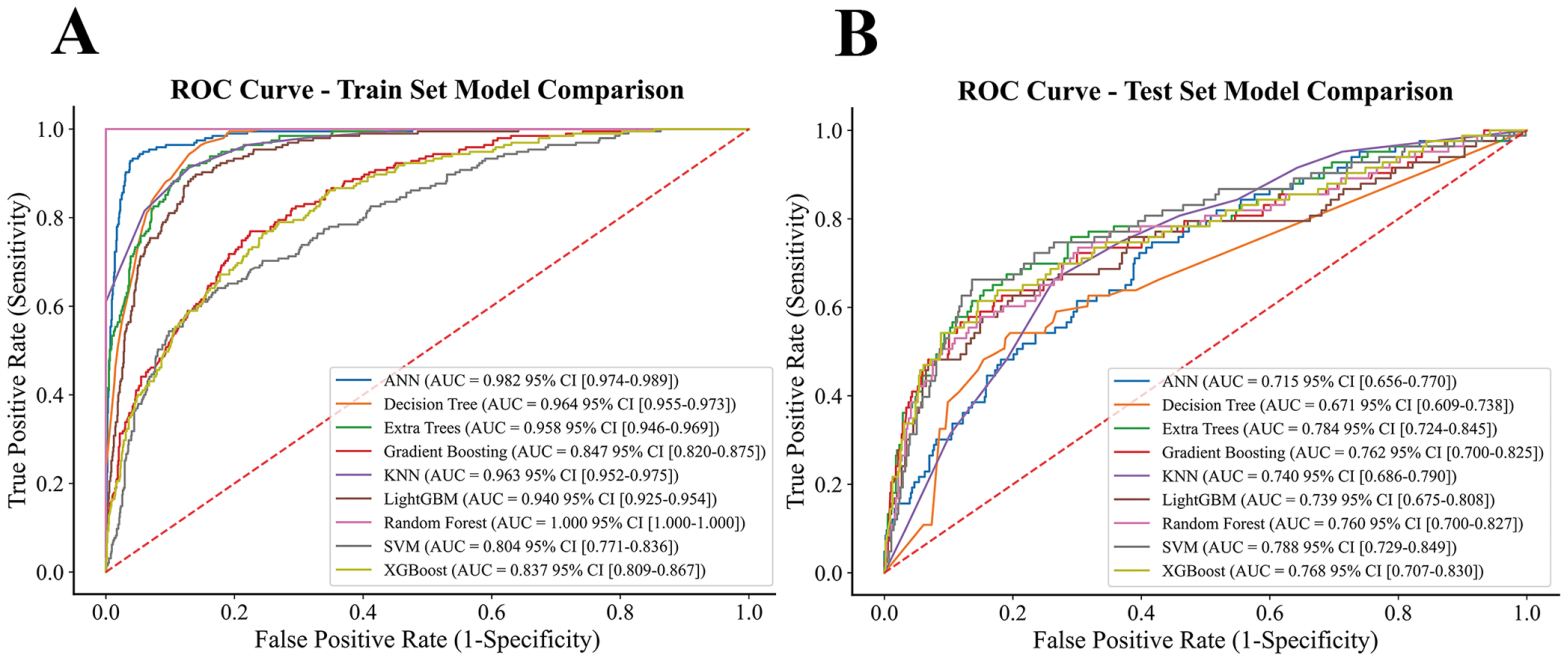
ROC and DCA curves for nine machine learning models. **(A)** Training set. **(B)** Testing set.

**Figure 5:**
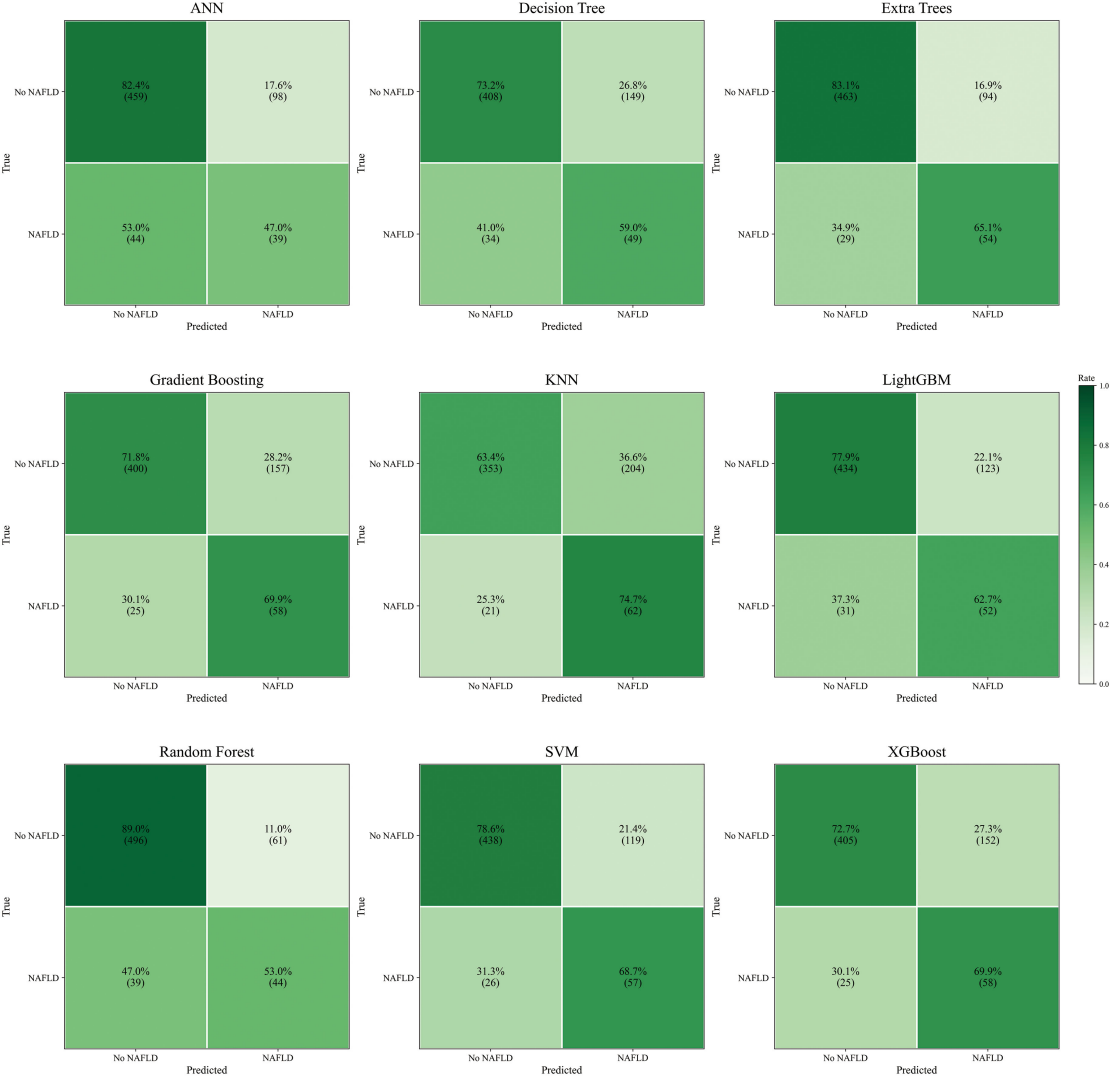
Confusion matrices of nine machine learning models.

**Figure 6:**
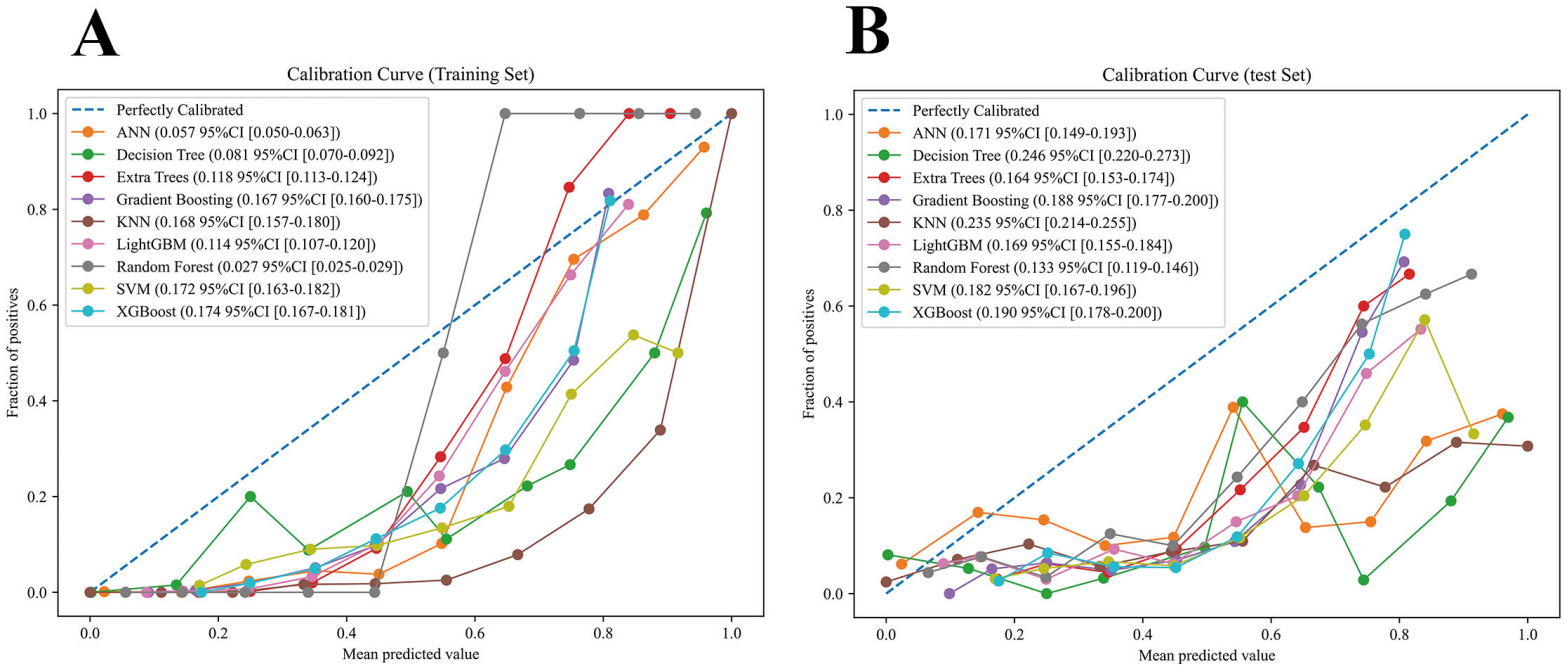
Calibration curves of nine machine learning models. **(A)** Training set. **(B)** Testing set.

**Figure 7:**
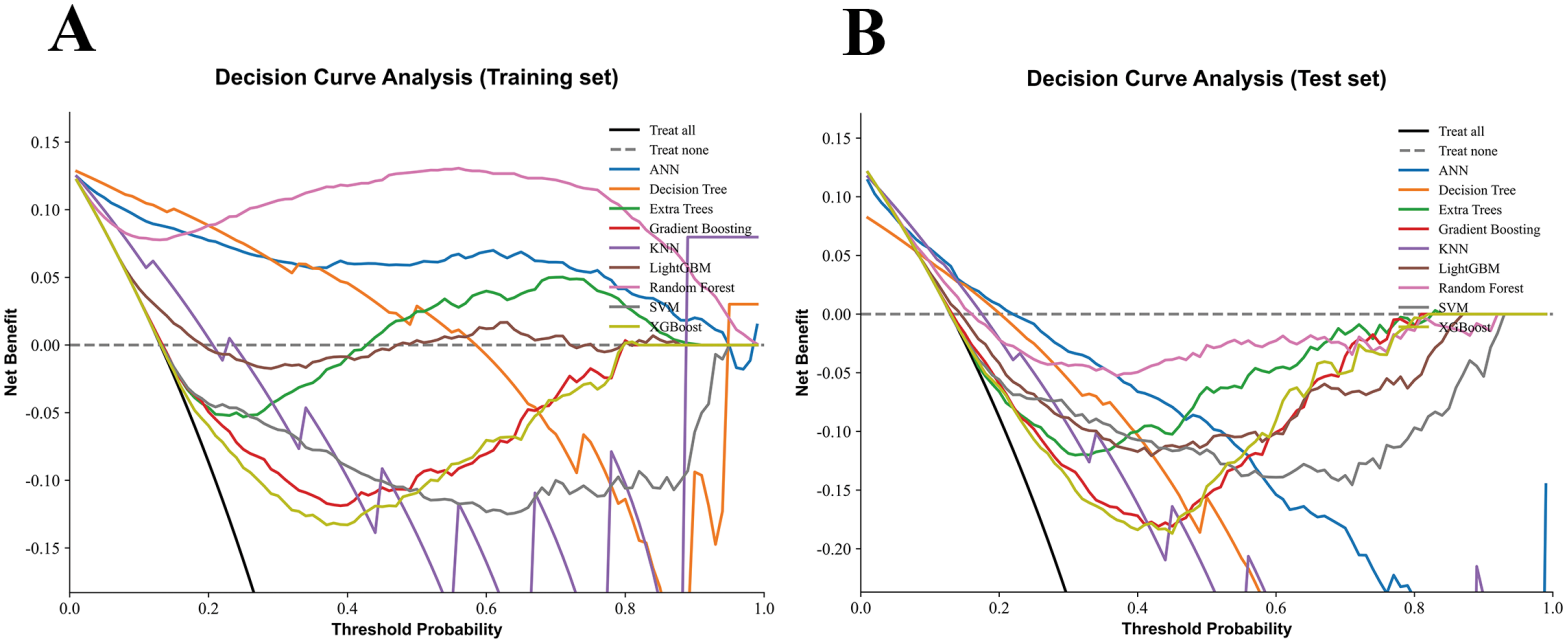
Decision curve analysis (DCA) of nine machine learning models.

Nine machine learning models were developed and evaluated using the top ten selected features. **Figure 4** displays the ROC curves for all models in both training and testing datasets. In the training set, AUCs ranged from 0.804 (SVM) to 1.000 (RF), with most models achieving values above 0.90. In the independent test set, AUCs were more modest, ranging from 0.671 (DT) to 0.788 (SVM). Specifically, the AUCs (95% CI) for each model were: ANN, 0.715 (0.656–0.770); DT, 0.671 (0.609–0.738); ET, 0.784 (0.724–0.845); GB, 0.762 (0.700–0.825); KNN, 0.740 (0.686–0.790); LGBM, 0.739 (0.675–0.808); RF, 0.760 (0.700–0.827); SVM, 0.788 (0.729–0.849); and XGBoost, 0.768 (0.707–0.830). Detailed classification metrics including accuracy, sensitivity, specificity, precision, F1-score, and Kappa are summarized in **Table 2**. **Figure 5** shows the confusion matrices of the nine models on the testing set. The proportion of correctly classified non-NAFLD participants ranged from 63.4% (KNN) to 83.1% (ET), while correct identification of NAFLD cases varied between 41.0% (DT) and 74.7% (KNN). Models such as ET, RF, GB, and SVM demonstrated relatively high true negative rates, whereas KNN and GB achieved comparatively higher true positive rates. Detailed counts and proportions for each cell of the confusion matrices are displayed in **Figure 5**. **Figure 6** presents the calibration curves for all models. Most algorithms showed acceptable agreement between predicted and observed probabilities, though calibration varied. In the test set, Brier scores ranged from 0.074 to 0.246, with ET, LGBM, GB, and XGBoost showing closer alignment to the reference line, while SVM and DT deviated more substantially. **Figure 7** displays the decision curve analysis (DCA). Across a wide range of threshold probabilities, tree-based ensemble models generally achieved higher net clinical benefit than single classifiers. Among the nine models evaluated, the Extra Trees (ET) algorithm achieved the best overall performance (AUC = 0.784, Brier score = 0.074) with the highest net clinical benefit. ET was therefore selected as the optimal model for subsequent comparison with traditional metabolic indicators.

**Table 2:** Performance comparison of nine machine learning models for NAFLD prediction in the testing set.

Model	AUC	ACC	SEN	PRE	F1 Score	Kappa
ANN	0.715	0.778	0.470	0.285	0.355	0.230
DT	0.671	0.673	0.602	0.221	0.324	0.165
ET	0.784	0.808	0.651	0.365	0.468	0.361
GB	0.762	0.716	0.699	0.270	0.389	0.249
KNN	0.740	0.648	0.747	0.233	0.355	0.196
LightGBM	0.739	0.759	0.627	0.297	0.403	0.276
RF	0.760	0.844	0.530	0.419	0.468	0.378
SVM	0.788	0.747	0.723	0.302	0.426	0.297
XGBOOST	0.768	0.723	0.699	0.276	0.396	0.258

Models include ANN (artificial neural network), DT (decision tree), ET (extra trees), GB (gradient boosting), KNN (k-nearest neighbors), LightGBM (Light Gradient Boosting Machine), RF (random forest), SVM (support vector machine), and XGBoost (eXtreme Gradient Boosting). Metrics: AUC, area under the curve; ACC, accuracy; SEN, sensitivity; PRE, precision.

**Figure 8:**
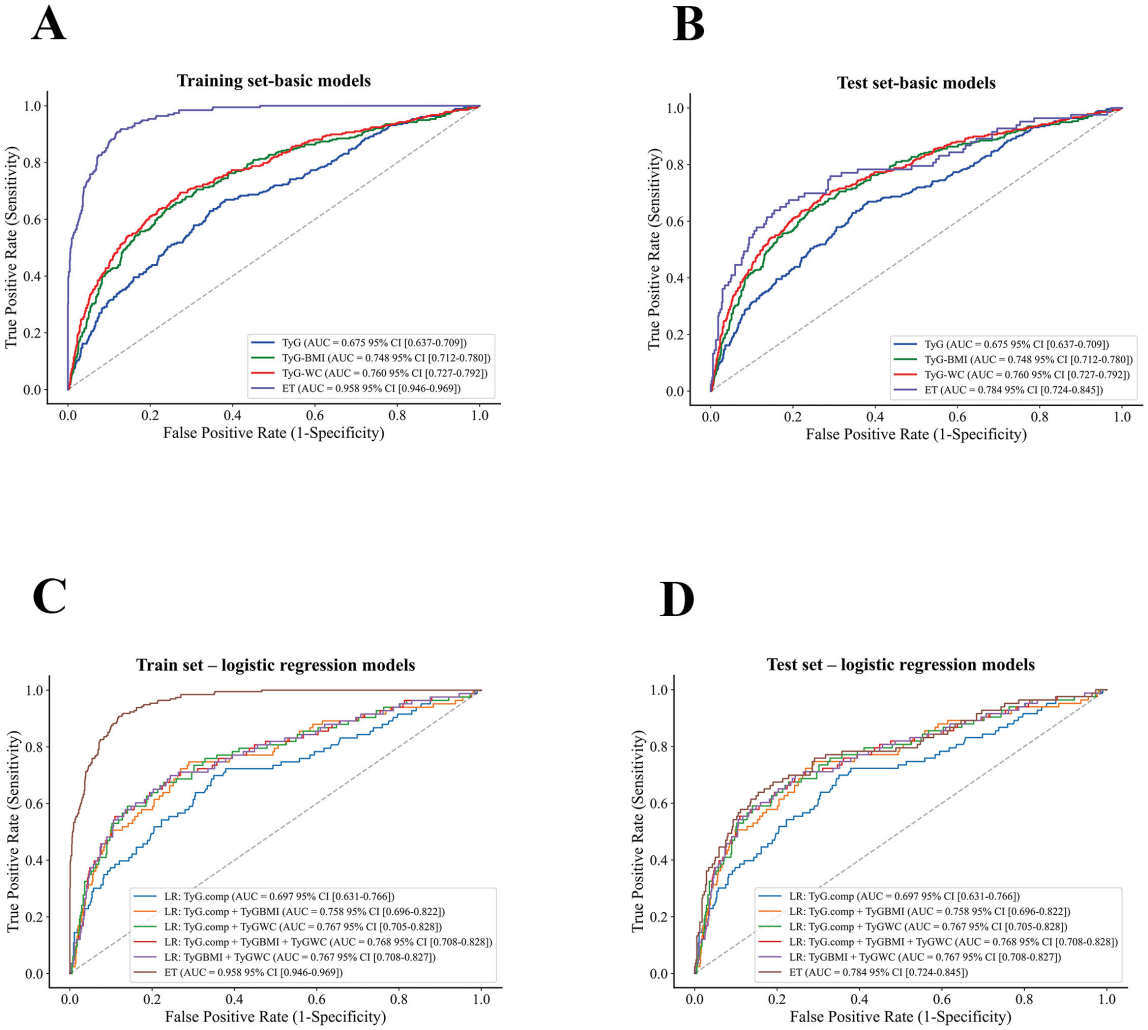
ROC curves comparing the Extra Trees (ET) model with TyG-based logistic regression models. **(A)** Training set – basic models. **(B)** Test set – basic models. **(C)** Training set – logistic regression models. **(D)** Test set – logistic regression models.

**Figure 8** and **Tables 3**, **4** compare the ET model with logistic regression models based on the TyG index and its derivatives. In the training set, AUCs ranged from 0.675 (TyG) to 0.958 (ET), while in the test set they ranged from 0.675 (TyG) to 0.784 (ET). Among the TyG-based models, derivatives such as TyG-BMI (AUC = 0.748), TyG-WC (AUC = 0.760), and multi-feature combinations (AUC up to 0.768) showed improved discrimination over TyG alone but remained inferior to ET. On the test set, the ET model achieved higher overall accuracy (0.773), precision (0.324), and Kappa (0.320), reflecting more balanced classification. In contrast, TyG-derived models often reached higher sensitivity (e.g., TyG-WC = 0.823) but at the expense of reduced precision and agreement, suggesting a tendency to overclassify positive cases. In summary, the ET model outperformed commonly used TyG-based traditional indicators, providing more reliable and balanced predictive performance.

**Table 3:** Performance comparison between the ET model and TYG-based indicators in the testing set.

Model	AUC	ACC	SEN	PRE	F1 Score	Kappa
TYG	0.675	0.653	0.633	0.206	0.311	0.153
TYG.BMI	0.748	0.720	0.722	0.266	0.389	0.255
TYG.WC	0.760	0.714	0.823	0.278	0.415	0.283
ET	0.784	0.773	0.687	0.324	0.440	0.320

ET, extra trees; TyG, triglyceride-glucose index; BMI, body mass index; WC, waist circumference. Metrics as in **Table 2**.

**Table 4:** Performance comparison of the ET model and logistic regression models based on TYG and its derived indices in the testing set.

Model	AUC	ACC	SEN	PRE	F1 Score	Kappa
TYG	0.697	0.653	0.633	0.206	0.311	0.153
TYG+TYG.BMI	0.758	0.736	0.684	0.273	0.390	0.259
TYG+TYG.WC	0.767	0.791	0.658	0.327	0.437	0.326
TYG+TYG.BMI+TYG.WC	0.768	0.797	0.696	0.342	0.458	0.351
TYG.BMI+TYG.WC	0.767	0.786	0.734	0.333	0.458	0.348
ET	0.784	0.773	0.687	0.324	0.440	0.320

ET, extra trees; TyG, triglyceride-glucose index; BMI, body mass index; WC, waist circumference. Metrics as in **Table 2**.

**Figure 9:**
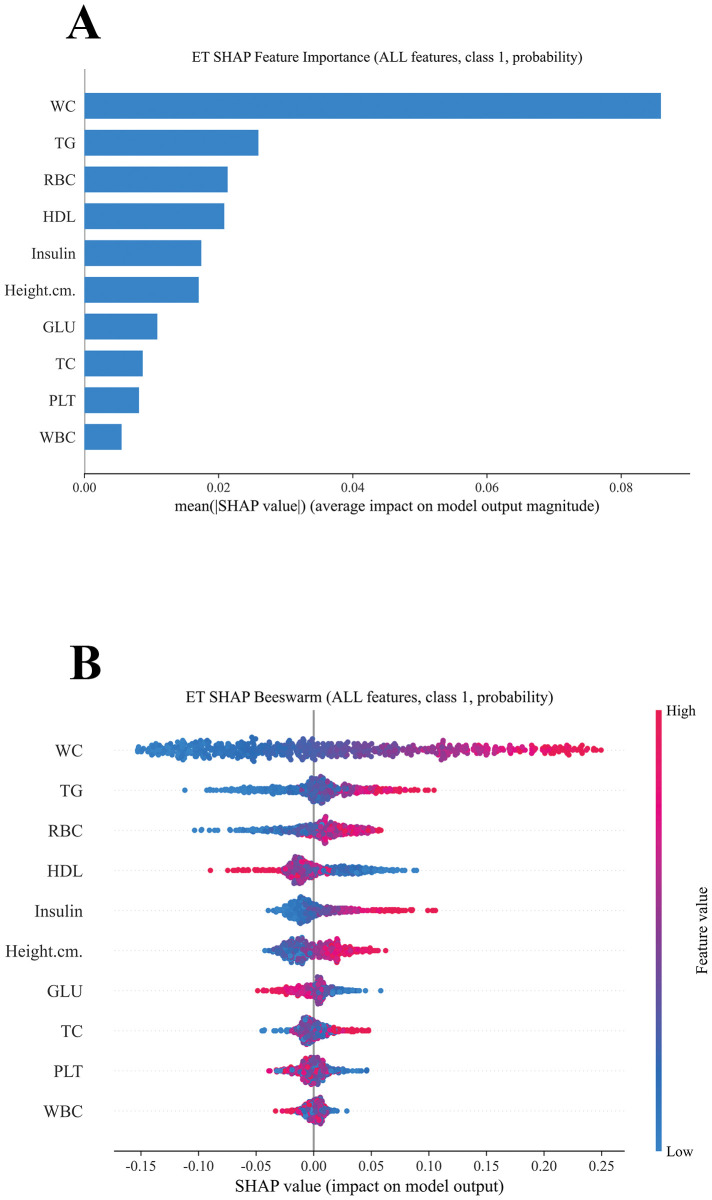
**(A)** Ranked feature importance of the ET model. **(B)** SHAP summary (beeswarm) plot showing direction and magnitude of feature contributions.

**Figure 10:**
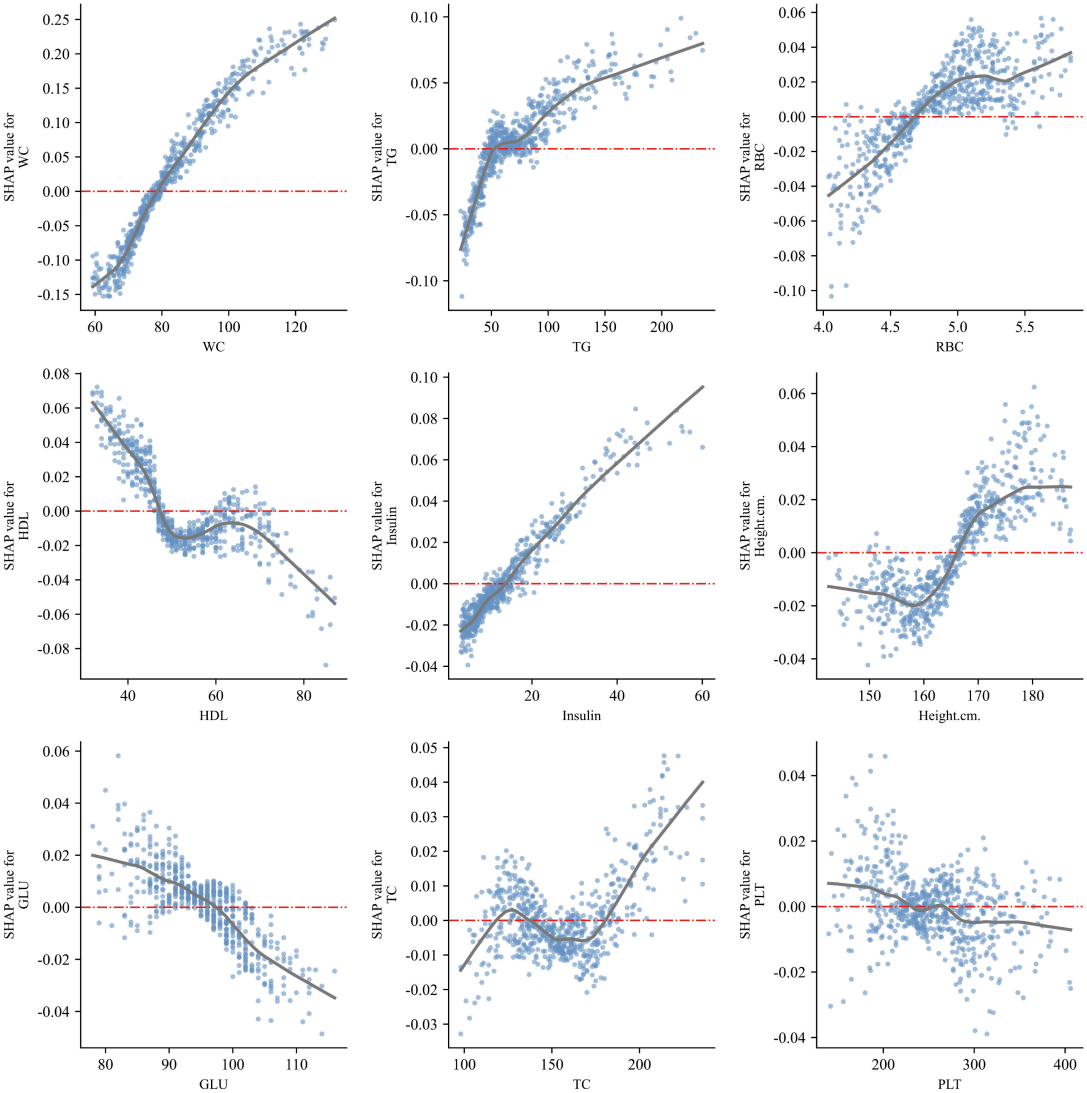
SHAP dependence plots for the nine most influential variables, illustrating nonlinear associations.

**Figure 11:**
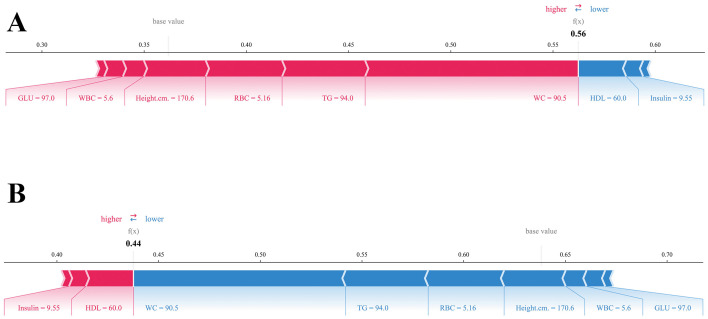
SHAP force plots for the first test-set participant: **(A)** predicted probability for NAFLD = 0.56; **(B)** predicted probability for non-NAFLD = 0.44.

SHAP analysis confirmed waist circumference (WC), triglycerides (TG), insulin, red blood cell count (RBC), and HDL as the most influential predictors of adolescent NAFLD, with glucose and platelet count also contributing (**Figure 9**). The SHAP summary plot (**Figure 9B**) demonstrated how higher WC, TG, and insulin levels increased risk, whereas higher HDL was protective. Dependence plots (**Figure 10**) further revealed nonlinear threshold effects, such as sharp risk increases at elevated WC and TG. At the individual level, SHAP force plots (**Figure 11**) decomposed predictions into feature-specific contributions, estimating, for example, a NAFLD probability of 0.56 versus 0.44 for non-NAFLD in a representative case. These visualizations provide clinically interpretable insights at both population and patient levels. Notably, the online risk prediction tool developed in this study adopts a similar framework: users input individual clinical and laboratory values, and the system generates a SHAP-like explanation of their personalized NAFLD risk. Together, these visualizations enhance both population-level interpretation and individual-level applicability.

## 4 Discussion

In this study, we developed predictive models for adolescent NAFLD using NHANES data (2011-2020) and nine supervised algorithms. To address class imbalance (13% vs. 87%), SMOTE was applied during training. Feature selection identified WC, TG, insulin, HDL, and RBC count as the most influential predictors. While these are established risk factors, the ML framework added value by quantifying their relative contributions, capturing nonlinear effects, and enabling individualized prediction through SHAP analysis. Comparative evaluation showed that the Extra Trees (ET) model outperformed commonly used TyG-based indices and achieved the most balanced performance across discrimination, accuracy, and agreement metrics. Finally, we deployed the ET model as an online risk calculator to support practical application in adolescent NAFLD screening.

Over the past decade, the prevalence of NAFLD in the United States has increased from 34.4% to 38.1%, paralleling the rise in obesity and type 2 diabetes mellitus (T2DM) (20). Among children and adolescents with obesity, the prevalence is approximately 36.1% and is expected to rise further with the global obesity epidemic (21). Pediatric NAFLD often persists into adulthood and can progress to fibrosis, cirrhosis, or other complications, underscoring the need for early detection. However, the optimal timing, frequency, and modality of screening remain unclear, and current evidence in adolescents is limited. While liver biopsy is the diagnostic gold standard, its invasiveness and cost preclude large-scale use (22). Conventional ultrasonography is more practical but has limited sensitivity for mild steatosis (23), the controlled attenuation parameter (CAP) has been proposed as a first-line screening tool in the general population, providing a more objective and quantifiable assessment of hepatic fat and serving as a useful adjunct to conventional ultrasound (24). However, its performance appears less reliable in pediatric populations, likely due to differences in body habitus and abdominal fat distribution that compromise imaging accuracy (25). Magnetic resonance imaging–derived proton density fat fraction (MRI-PDFF) provides the most accurate noninvasive quantification of hepatic fat and performs well in children, but its high cost and technical demands restrict routine use. Consequently, recent research has emphasized the need for reliable serum biomarkers for large-scale adolescent NAFLD screening (26). In addition, recent studies have applied machine learning specifically to pediatric and adolescent populations, including an NHANES-based adolescent model and a multi-algorithm pediatric study, both of which reported encouraging predictive performance and provided interpretable insights into feature contributions (27, 28).

Using the LGBM algorithm, we initially ranked variables by feature importance and identified the top ten predictors: WC, insulin, TG, PLT, Height, GLU, WBC, TC, RBC, and HDL. These factors are well documented in adults—WC as the strongest body composition predictor of NAFLD (29, 30), insulin resistance as a central drive (31), and TG accumulation as a key pathological hallmark (32). Platelet and red blood cell indices have also been implicated in liver injury and repair processes (33–36). Although these associations are established, their relative contributions and interactions in adolescents remain understudied. To ensure robustness, we validated the LGBM-based selection with a consensus strategy combining L1-logistic regression, Boruta, and permutation importance, which consistently highlighted overlapping predictors. This confirmed that our feature selection was not biased toward tree-based methods and provided a stable foundation for subsequent model development.

Comparative evaluation of nine supervised algorithms demonstrated that the Extra Trees (ET) model achieved the most consistent overall performance across discrimination, classification, calibration, and clinical utility. In ROC analysis, ET yielded the highest AUC in both training and testing sets, reflecting strong discriminative ability compared with ensemble and non-ensemble algorithms. Confusion matrix results further confirmed its balanced classification, with markedly higher sensitivity for NAFLD detection than most counterparts, while maintaining high overall accuracy. In terms of calibration, the ET model produced the lowest Brier score and curves closely aligned with the reference line, indicating reliable probability estimates. Decision curve analysis (DCA) also showed that ET consistently provided the greatest net clinical benefit across a wide range of threshold probabilities, outperforming the other eight models. Taken together, these findings indicate that ET offered the most robust balance of discrimination, reliability, and clinical applicability, supporting its selection as the optimal algorithm for subsequent comparison with traditional metabolic indices. Consistent with previous studies, ensemble tree-based methods such as ET and RF have repeatedly shown strong generalization in predicting chronic diseases, including NAFLD (37–39). Our study extends this evidence to adolescents, representing the first application of ET in this population.

When compared with logistic regression models based on the TyG index and its derivatives, the ET model consistently demonstrated superior predictive balance. Although TyG-derived models—particularly TyG-WC (sensitivity = 0.823) and multi-feature combinations (AUC up to 0.768)—achieved higher sensitivity than ET, this came at the cost of lower precision and overall accuracy, reflecting a tendency to overclassify positive cases. By contrast, ET maintained the highest AUC (0.784), along with better precision (0.324) and agreement (Kappa = 0.320), offering a more reliable performance profile. These results suggest that while TyG indices capture important aspects of insulin resistance, their limited dimensionality constrains their predictive value. ET, by integrating complex nonlinear interactions, provides superior discrimination and more balanced performance, making it a more appropriate tool for adolescent NAFLD risk prediction.

SHAP analysis confirmed waist circumference (WC), triglycerides (TG), insulin, red blood cell count (RBC), and HDL as the most influential predictors of adolescent NAFLD, with glucose and platelet count also contributing (**Figure 9**). Beyond confirming known risk factors, the model quantified their relative importance and revealed nonlinear effects. Dependence plots indicated that higher WC and TG sharply increased risk, while elevated HDL exerted a protective but nonlinear effect (**Figure 10**). At the individual level, SHAP force plots illustrated how multiple features jointly shaped predictions, with WC and TG driving positive contributions and HDL and insulin reducing risk (**Figure 11**). These results provide clinically interpretable insights at both population and patient levels.

From a clinical perspective, the ET-based model is practical as it uses routinely available anthropometric and laboratory measures, enabling scalable screening in adolescents. The identification of nonlinear thresholds for WC, TG, and HDL offers actionable cutoffs for early intervention, while the accompanying online tool provides individualized risk estimates to support tailored prevention and patient engagement.

This study has several limitations. It was based on cross-sectional NHANES data, restricting causal inference, and NAFLD diagnosis relied on biochemical indicators rather than liver biopsy, which may have caused misclassification. Although multiple feature selection strategies were applied, important genetic or environmental factors might have been overlooked. In addition, the absence of external cohort validation and the U.S.-only adolescent sample limit generalizability to other populations. Nonetheless, the study’s strengths include the use of a large, nationally representative dataset with rigorous inclusion criteria, adjustment for key confounders, and the development of an accessible online prediction tool, together supporting its reliability and clinical relevance.

## 5 Conclusion

The machine learning model developed using the Extra Trees algorithm in this study demonstrates superior predictive performance for identifying adolescents at risk of NAFLD. Based on this model, an interactive web-based prediction tool was constructed, enabling clinicians to rapidly and conveniently estimate individual NAFLD risk using routine clinical indicators. This model not only improves early identification and risk stratification of NAFLD in youth populations but also has the potential to reduce unnecessary imaging examinations and laboratory testing, ultimately supporting cost-effective and personalized preventive strategies in clinical practice.

## Data Availability

The original contributions presented in the study are included in the article/**Supplementary Material**. Further inquiries can be directed to the corresponding author.
