# Correction: From traditional metabolic markers to ensemble learning: comparative application of machine learning models for predicting NAFLD risk in adolescents

**DOI:** 10.3389/fendo.2026.1763989

**Published:** 2026-02-05

**Authors:** Chenming Zhang, Bin Niu, Rong Wang, Liaoyun Zhang

**Affiliations:** 1 Academy of Medical Sciences, Shanxi Medical University, Taiyuan, China; 2 Department of Infectious Diseases, The First Hospital of Shanxi Medical University, Taiyuan, China; 3 Graduate School, Shanxi Medical University, Taiyuan, China

**Keywords:** machine learning, non-alcoholic fatty liver disease, adolescents, feature selection, public health

There was a mistake in **Tables 2**–**4** as published. **Tables 2**–**4** contained a formatting/layout issue: in each table, an extra blank table appeared above the intended populated table.

**Table 2:** Performance comparison of nine machine learning models for NAFLD prediction in the testing set.

Model	AUC	ACC	SEN	PRE	F1 Score	Kappa
ANN	0.715	0.778	0.470	0.285	0.355	0.230
DT	0.671	0.673	0.602	0.221	0.324	0.165
ET	0.784	0.808	0.651	0.365	0.468	0.361
GB	0.762	0.716	0.699	0.270	0.389	0.249
KNN	0.740	0.648	0.747	0.233	0.355	0.196
LightGBM	0.739	0.759	0.627	0.297	0.403	0.276
RF	0.760	0.844	0.530	0.419	0.468	0.378
SVM	0.788	0.747	0.723	0.302	0.426	0.297
XGBOOST	0.768	0.723	0.699	0.276	0.396	0.258

Models include ANN (artificial neural network), DT (decision tree), ET (extra trees), GB (gradient boosting), KNN (k-nearest neighbors), LightGBM (Light Gradient Boosting Machine), RF (random forest), SVM (support vector machine), and XGBoost (eXtreme Gradient Boosting). Metrics: AUC, area under the curve; ACC, accuracy; SEN, sensitivity; PRE, precision.

**Table 3:** Performance comparison between the ET model and TYG-based indicators in the testing set.

Model	AUC	ACC	SEN	PRE	F1 Score	Kappa
TYG	0.675	0.653	0.633	0.206	0.311	0.153
TYG.BMI	0.748	0.720	0.722	0.266	0.389	0.255
TYG.WC	0.760	0.714	0.823	0.278	0.415	0.283
ET	0.784	0.773	0.687	0.324	0.440	0.320

ET, extra trees; TyG, triglyceride-glucose index; BMI, body mass index; WC, waist circumference. Metrics as in **Table 2**.

**Table 4:** Performance comparison of the ET model and logistic regression models based on TYG and its derived indices in the testing set.

Model	AUC	ACC	SEN	PRE	F1 Score	Kappa
TYG	0.697	0.653	0.633	0.206	0.311	0.153
TYG+TYG.BMI	0.758	0.736	0.684	0.273	0.390	0.259
TYG+TYG.WC	0.767	0.791	0.658	0.327	0.437	0.326
TYG+TYG.BMI+TYG.WC	0.768	0.797	0.696	0.342	0.458	0.351
TYG.BMI+TYG.WC	0.767	0.786	0.734	0.333	0.458	0.348
ET	0.784	0.773	0.687	0.324	0.440	0.320

ET, extra trees; TyG, triglyceride-glucose index; BMI, body mass index; WC, waist circumference. Metrics as in **Table 2**.

The corrected **Tables 2**–**4** appear below.

The original version of this article has been updated.

