# Supplementary figures and images for: From traditional metabolic markers to ensemble learning: comparative application of machine learning models for predicting NAFLD risk in adolescents
Source: Front Endocrinol (Lausanne). 2025 Oct 29;16:1681686. doi: 10.3389/fendo.2025.1681686 (PMC12605207; doi:10.3389/fendo.2025.1681686)

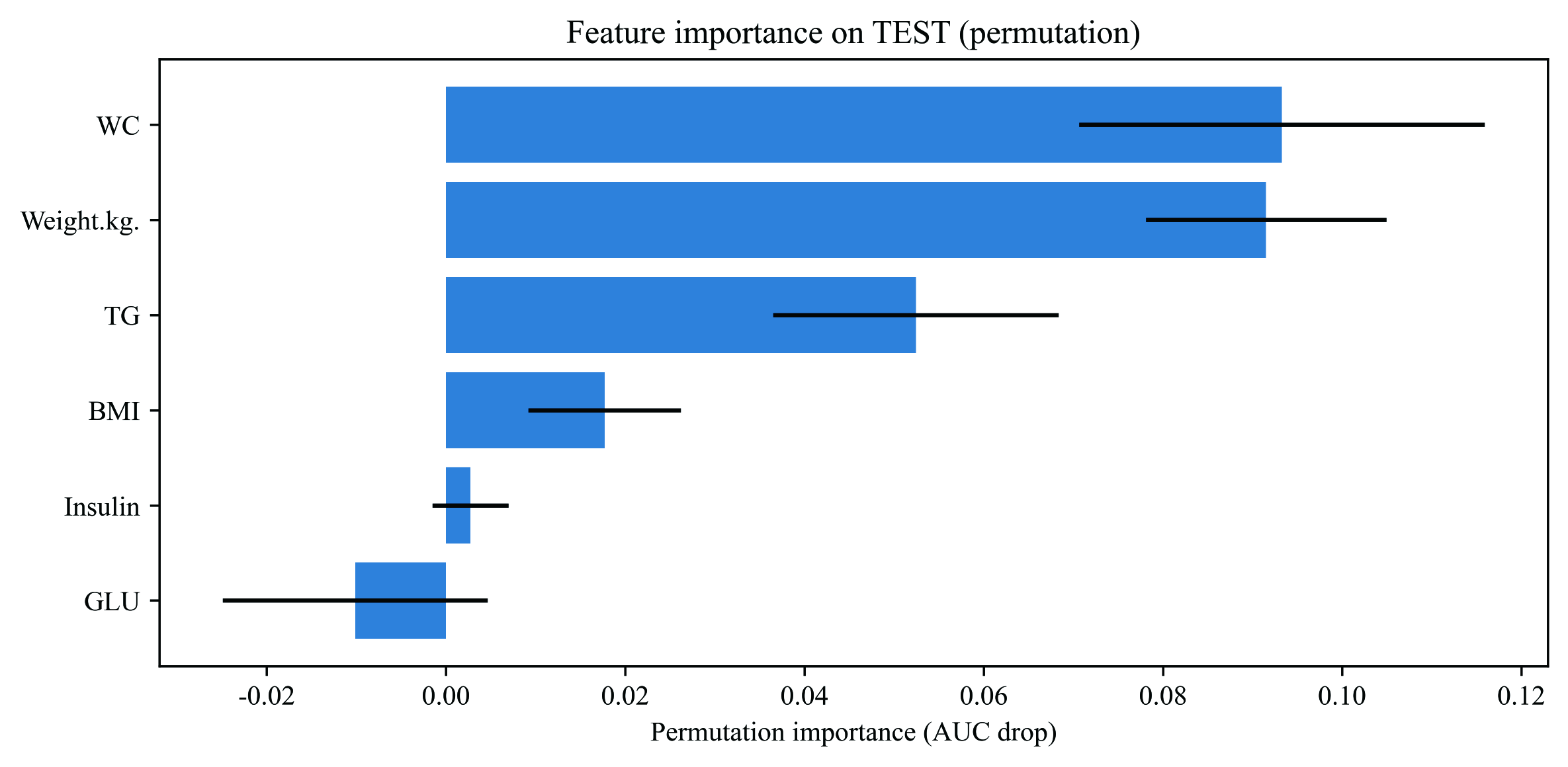

Supplement: Supplementary file 3 [file Image1.tif]

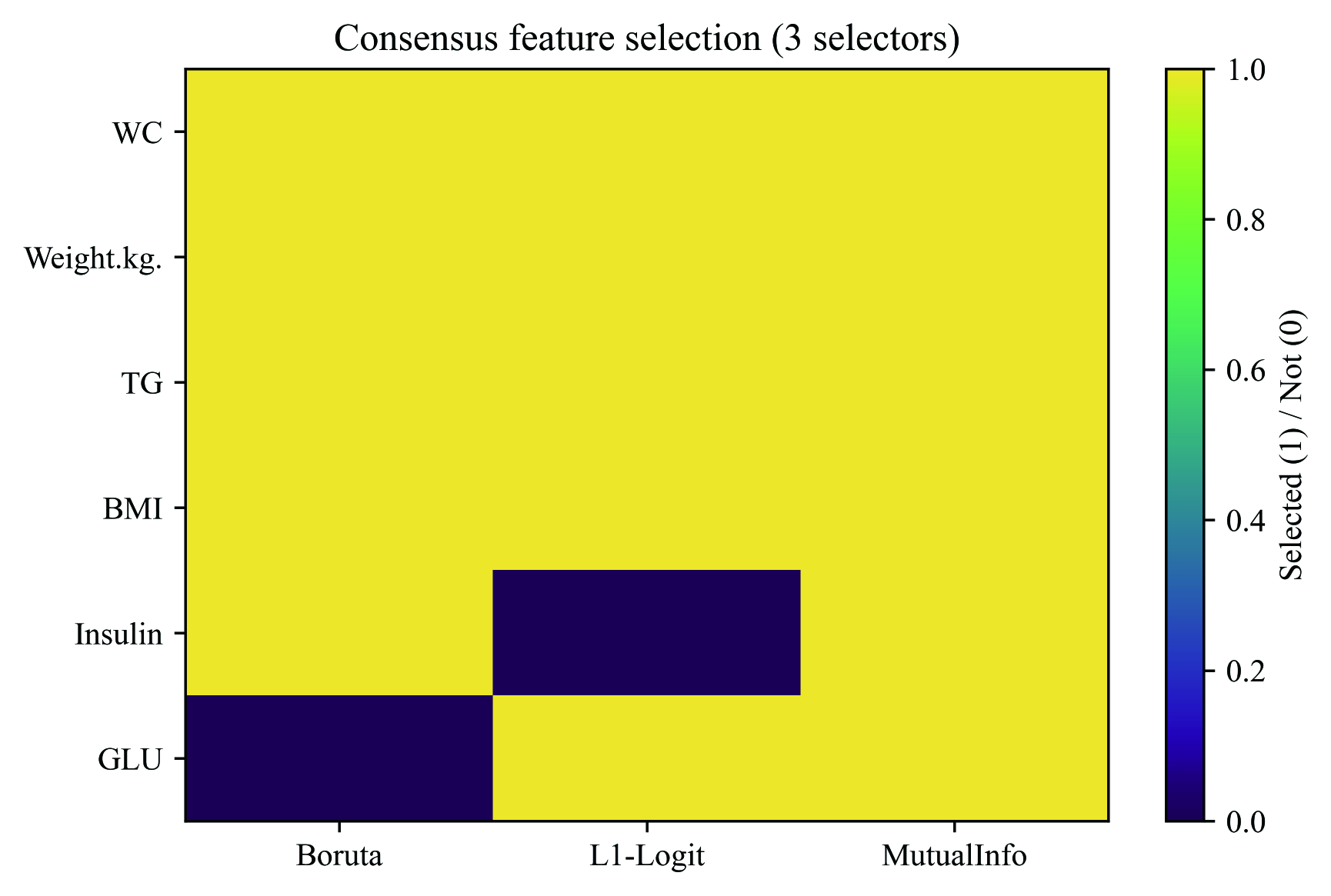

Supplement: Supplementary file 4 [file Image2.tif]
